# Hybrid fiber resonator employing LRSPP waveguide coupler for gyroscope

**DOI:** 10.1038/srep41146

**Published:** 2017-01-24

**Authors:** Guang Qian, Xing-Chang Fu, Li-Jiang Zhang, Jie Tang, Yi-Ran Liu, Xiao-Yang Zhang, Tong Zhang

**Affiliations:** 1 School of Instrument Science and Engineering, Key Laboratory of Micro-Inertial Instrument and Advanced Navigation Technology, Ministry of Education, Southeast University, Nanjing, 210096, China; 2 School of Electronic Science and Engineering, Joint International Research Laboratory of Information Display and Visualization, Southeast University, Nanjing, 210096, China; 3 Suzhou Key Laboratory of Metal Nano-Optoelectronic Technology, Suzhou Research Institute of Southeast University, Suzhou, 215123, China

## Abstract

Polarization error and temperature noise are two main limits to the performance of resonant fiber optic gyroscope (RFOG). To overcome these limits, we demonstrated a hybrid resonator consisting of a polymer-based long-range surface plasmon polariton (LRSPP) waveguide coupler and a silica fiber. Single-polarization property of LRSPP waveguide and the offsetting of the opposite thermo-optical characteristics between the polymer-based LRSPP waveguide and the silica fiber can effectively inhibit both the polarization error and the temperature noise of RFOG. The measured resonance spectrum of the hybrid resonator shows the absence of polarization noise. The temperature dependence of wavelength shift (TDWS) of resonator dropped to about 2 pm/°C, or even to 0 pm/°C with optimal structure, which dramatically improves the temperature stability of gyroscope system. In addition, the hybrid resonator also shows tremendous application potential in rate-grade and tactical-grade gyroscopes.

Resonant fiber optic gyroscope (RFOG) based on Sagnac effect has been considered as a promising candidate for the new-generation inertial navigation system^1^,^2^,^3^. In practice, its performance to date is still below expectation due to various effects that produce noises. Among them, the polarization-fluctuation induced drift, which mainly caused by the existence of dual eigen-states of polarization (ESOP), has been regarded as the dominant non-reciprocal noise of RFOG^4^. In addition, resonant wavelength of resonator is extremely sensitive to the fluctuation of ambient temperature, which makes it difficult to obtain a high-performance RFOG^5^,^6^.

To overcome these limits, efforts have been made during the past decades. Many resonator schemes have been proposed to reduce the polarization noise, including polarization-maintaining fiber (PMF) resonator employing polarization controller (PC) or fiber polarizer, PMF resonator with 90° polarization-axis rotated splice^7^, hollow-core photonic bandgap fiber (PBF) resonator^8^,^9^, single-mode fiber resonator composed of two parts of oppositely twisted fiber^10^ and etc. However, these schemes have some shortcomings. For example, the added polarization controller or fiber polarizer always requires high precision, and also seriously enlarges the gyroscope system. The resonators based on 90° polarization-axis rotated splice and two portions of oppositely twisted single-mode fiber have unavoidable fabrication error. The PBF-based resonator is usually high cost. In practice, almost all the proposed schemes can only reduce rather than completely estimate the polarization noise. In addition, these schemes are unable to remove the influence of temperature fluctuation on resonant wavelength, even the resonator formed by hollow-core PBF^11^. Therefore, a novel resonator scheme with characteristics of single ESOP, athermality and low cost is required to overcome the above bottlenecks of RFOG.

Long-range surface plasmon polaritons (LRSPP) waveguides is a new research hotspot of integrated optics^12^,^13^,^14^. This waveguide consists of nanometer-thin metal strips embedded in homogeneous dielectric environment. The guided mode is a mixed mode of electrons and photons propagating along a metal-dielectric interface, and is always transverse-magnetic (TM) polarized^15^,^16^,^17^. LRSPPs show good mode-matching to conventional optical fibers^18^, as well as simultaneous guiding of optical and electrical signals^19^,^20^. These advantages render LRSPP waveguides an outstanding platform for specialized applications in high-precision optics^13^,^21^,^22^,^23^,^24^,^25^.

This letter reports a hybrid resonator with special properties of single polarization and temperature insensitive to overcome the bottlenecks of RFOG. The resonator was formed by a polymer-based LRSPP waveguide coupler and a silica fiber. The unwanted ESOP is effectively eliminated by the single-polarization LRSPP waveguide. The TDWS of the resonator is heavily reduced by the temperature insensitive characteristic. This hybrid resonator provides a very efficient solution to simultaneously inhibit polarization noise and temperature noise in RFOG, and promotes the rate-grade and tactical-grade RFOGs to be practical.

## Results and Discussions

**Figure 1:**
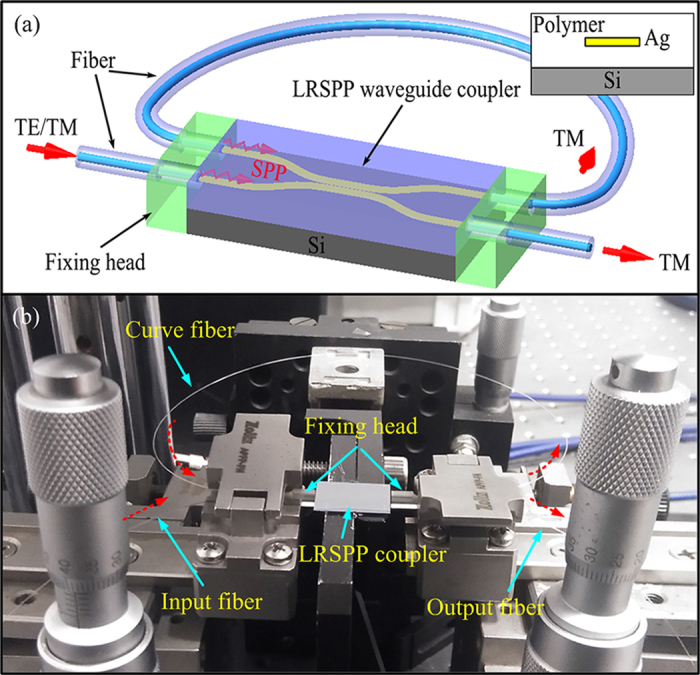
(**a**) Schematic of a hybrid resonator employing a LRSPP waveguide coupler. Inset: Cross-sectional sketch of the LRSPP waveguide. (**b**) Fabricated hybrid resonator placed on the testing platform.

Figure 1(a) shows the diagram of the hybrid resonator consisting of a curve fiber, an input fiber, an output fiber and a LRSPP waveguide coupler. The two ends of the curve fiber are spliced with the two ends of one of the LRSPP coupler arms. The input fiber and the output fiber are spliced with the two ends of the other arm. When a beam of light with both TE and TM polarizations couples into one arm of the LRSPP waveguide coupler from the input fiber, only the TM-polarized light can transmit through the LRSPP coupler and launches into the curve fiber. The single-polarization property of the hybrid resonator is ensured by the LRSPP waveguide coupler. In order to simplify the splices between the ends of fiber and coupler, the two ends of the curve fiber were respectively integrated with the ends of input and output fiber by two fixing heads. The space between same-side fiber ends is 250 μm that is same to the distance between the two arms of the coupler. Therefore, the integrated fiber ends can be precisely docked with the LRSPP coupler. The LRSPP waveguide consists of Si substrate, sliver strip and polymer cladding, as shown in the inset of Fig. 1(a). Figure 1(b) shows the fabricated hybrid resonator placed on the testing platform.

**Figure 2:**
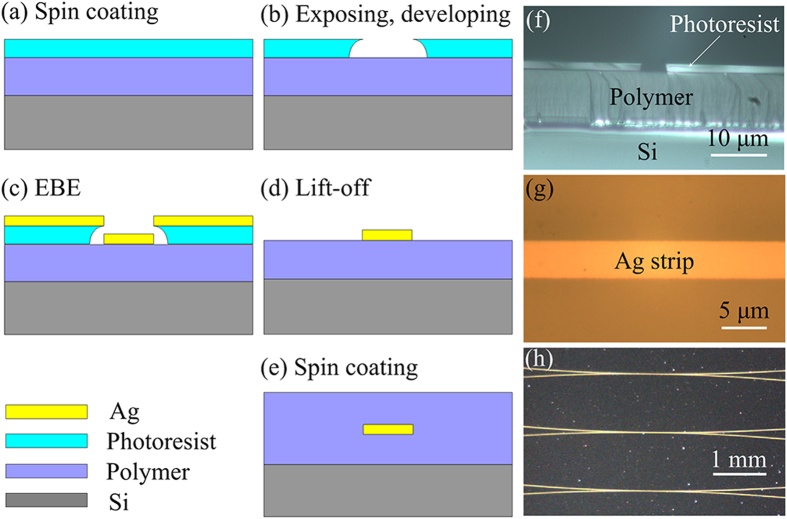
(**a**–**e**) Fabrication processes of LRSPP waveguide coupler. (**f**) Cross-section view of the trapezoidal groove of photoresist made by process (**b**). (**g**) Optical micrograph of sliver stripe in LRSPP waveguide. (**h**) Photograph of sectional LRSPP waveguide coupler.

Figure 2(a–e) show the main fabrication processes of LRSPP waveguide coupler, including spin coating, exposing, developing, electron beam evaporation (EBE) and lift-off. In order to ensure the low propagation loss, silver stripe with a width of about 4 μm and a thickness of about 10 nm was used as the core of the LRSPP waveguide. Low-loss polymer material, photo-active UV curable fluorinated resins based on acrylate, was used to make the waveguide cladding (n = 1.44 at 1550 nm). Additionally, the total thickness of the polymer cladding was designed to be about 40 μm. The silver strip was embedded in the polymer cladding. The detail fabrication processes of the LRSPP waveguide coupler was given in the method section. Figure 2(f) shows the cross-section of the photoresist pattern after process (b). A trapezoidal groove structure is very helpful for the latter lift-off process. Figure 2(g) shows the top view of the sliver strip under optical microscope. Figure 2(h) shows part of the LRSPP waveguide coupler.

**Figure 3:**
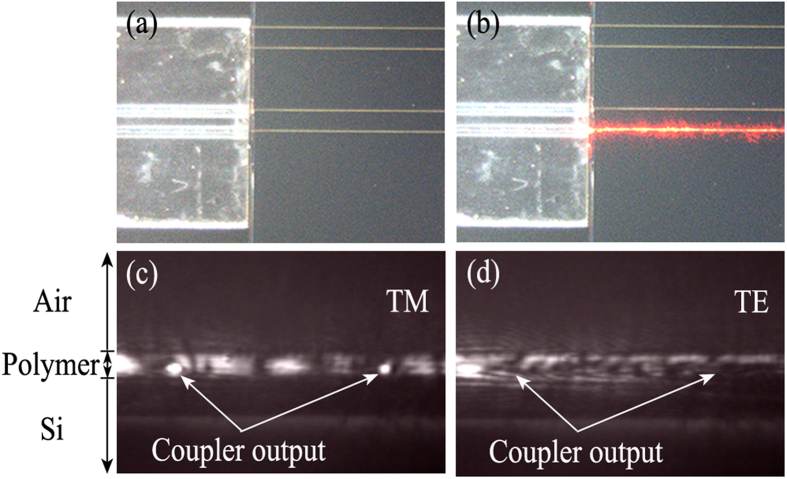
(**a**) and (**b**) Camera images of the alignment between the LRSPP waveguide coupler and the fibers. Camera images of one LRSPP waveguide coupler output for TM polarization (**c**) and TE polarization (**d**), showing single-mode output and no measurable output from the waveguide end-facet, respectively.

In order to study the polarization characteristics of the hybrid resonator, we first measured the polarization dependence of the LRSPP waveguide coupler at 1550 nm by an infrared CCD. Before the measurement, the two fibers in the fixing head must be aligned with the two arms of the LRSPP coupler respectively, as shown in Fig. 3(a). In order to align conveniently and accurately, 632.8 nm laser was used. When the silver strip was lighted by the red light, as shown in Fig. 3(b), the fiber was aligned with the arm of LRSPP waveguide coupler. Figure 3(c) and (d) show the camera images of the LRSPP waveguide coupler output for transverse magnetic (TM) polarization and transverse electric (TE) polarization, respectively. It is clearly shown that TM-polarized light can pass the LRSPP waveguide coupler, and TE-polarized light is blocked. This is just contributed by the TM-polarized property of LRSPP mode. In other words, in the hybrid resonator, only the TM-polarized component of the input light can propagates through the coupler and launches into the fiber resonator. The TE-polarized component is filtered out of the resonator. The LRSPP waveguide coupler plays the role of a polarizer to ensure the single-polarization in the hybrid resonator. Therefore, the input light can only excite one ESOP in the resonator, and with a result of one resonance dip.

**Figure 4:**
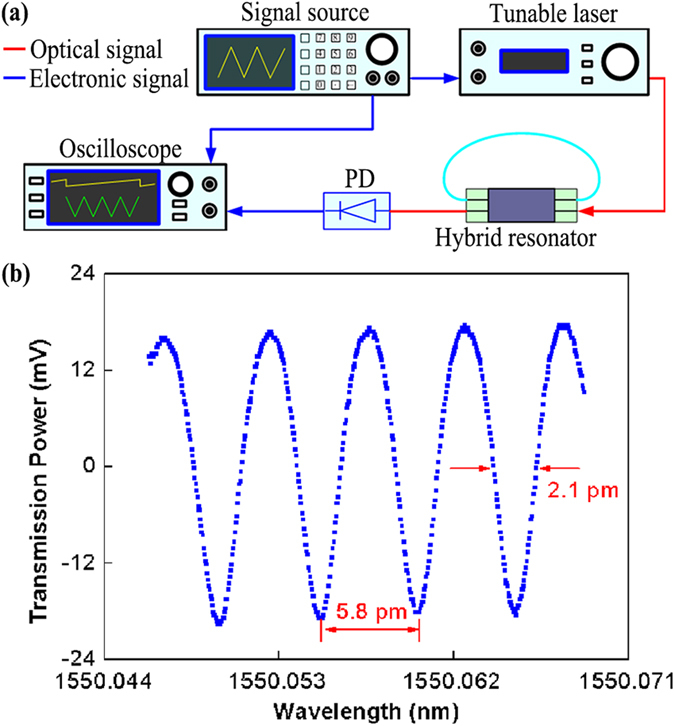
(**a**) Measurement setup for the hybrid resonator. (**b**) Measured transmission spectrum of a hybrid resonator formed by a 1.5-cm-long LRSPP coupler and a 30-cm-long SM fiber.

To realize the single-ESOP hybrid resonator, many kinds of fiber can be used, including single-mode fiber, single-polarization optical fiber, polarization-maintaining fiber, and photonic crystal fiber. In order to measure the single-polarization property, a signal source with sawtooth wave, a tunable laser of 1550 nm, a photodetector and an oscilloscope were used. The measurement setup is shown as Fig. 4(a). Figure 4(b) shows the measured resonance spectrum of a hybrid resonator formed by a 1.5-cm-long LRSPP coupler and a 30-cm-long single-mode fiber. Note that this set of dips show high degree of symmetry and lack of any unwanted polarization dips.

For the traditional RFOG resonators, there are always two ESOPs in the resonator changing with external factors, such as temperature, mechanical stress and etc. The mismatch between the input SOP and the ESOP in the resonator is the basic cause of producing polarization error in RFOG. The error is generally estimated by^4^


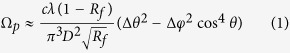



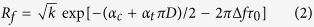


where *c, λ, D, k, α*_*c*_, *α*_*t*_, Δ*f* and *τ*_0_ are the light velocity in vacuum, the wavelength of the light source, the diameter of the resonator, the power coupling ratio of the coupler, the loss of the coupler, the loss of the resonator, the spectrum width of the light source and the integration time, respectively. The parenthetical section of 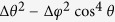 in Eq. (1) is generated by the mismatch of the input SOP 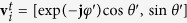 to one ESOP in the resonator 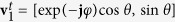, defined as 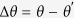 and 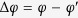. From Eq. (1) and Eq. (2), we can see that the value of Ω_*p*_ is determined by *λ, D, k, α*_*c*_, *α*_*t*_, Δ*f, τ*_0_ and the mismatch degree of the SOPs. According to Eq. (1) and Eq. (2), the mismatch Δ*θ* must be controlled to be <0.006° to achieve the high performance gyroscope for the inertial navigation system whose resolution must be <10^−7^ rad/s^4^. Therefore, the unstable two ESOPs is the bottleneck for high sensitivity RFOG. To overcome this, single-polarization RFOG resonator has been regarded as one of the most effective way^26^,^27^. The hybrid resonator proposed in this work provides an innovative and simple way to realize single-polarization RFOG resonator. In this resonator, there is no mismatch between SOPs because there is only one polarization mode in the resonator. Compared with the reported single-polarization RFOG resonators^7^,^26^,^27^, it shows remarkable advantage of exciting single ESOP in the RFOG resonator without fabrication error due to the TM-pass property of LRSPP waveguide coupler, also does not need assistive devices such as polarization controller, fiber polarizer and etc.

In addition, the hybrid resonator also shows potential advantages of low temperature dependence. Athermal resonator can be realized when the lengths of the LRSPP coupler and the ring fiber are well matched. Suppose the effective refraction index of fiber, the real part of the effective mode index of the LRSPP waveguide, the length of the curve fiber and the length of LRSPP waveguide coupler are denoted as *n*_*1*_, *N*_*eff(R*)_, *L*_*1*_ and *L*_*2*_, respectively. A resonant wavelength of the hybrid resonator obeys the following relationship.


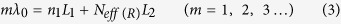


where *λ*_*0*_ is the resonant wavelength that is a function of temperature *T*. The temperature dependence of wavelength shift (TDWS) can be described by


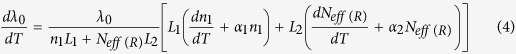


where *α*_*1*_ and *α*_*2*_ represent the thermal expansion coefficient (TEC) of the silica fiber and the Si substrate of the LRSPP waveguide, respectively. The thermo-optic coefficient (TOC), TEC and effective refractive index of silica fiber are obtained from ref. 28. The refractive index of the used polymer in the LRSPP coupler is 1.44, and the TOC of the polymer is about −1.86 × 10^−4^. The temperature dependence of the effective complex refractive index of silver, *N*_*Ag*_(T) = *N*_*r*_(T) + *iN*_*i*_(*T*), are *N*_*r*_(*T*) = 0.059 + 1.55 × 10^−3^ *T*, N_*i*_(*T*) = 10.93 − 3.37 × 10^−4^ *T* − 3.34 × 10^−7^ *T*^2^ at 1550 nm^29^. Using these temperature dependent refractive indices, we numerically calculated effective mode index *N*_*eff*_ of the LRSPP waveguide at different temperatures. These results show that the real parts of the effective mode index *N*_*eff(R*)_ vary approximately linearly with temperature changes. By calculating, we got the *dN*_*eff(R*)_/*dT* of LRSPP waveguide is about −1.859 × 10^−4^ that is approximate to the TOC of the polymer cladding. It indicates that the *dN*_*eff(R*)_/*dT* of LRSPP waveguide is mainly determined by the TOC of cladding polymer.

**Figure 5:**
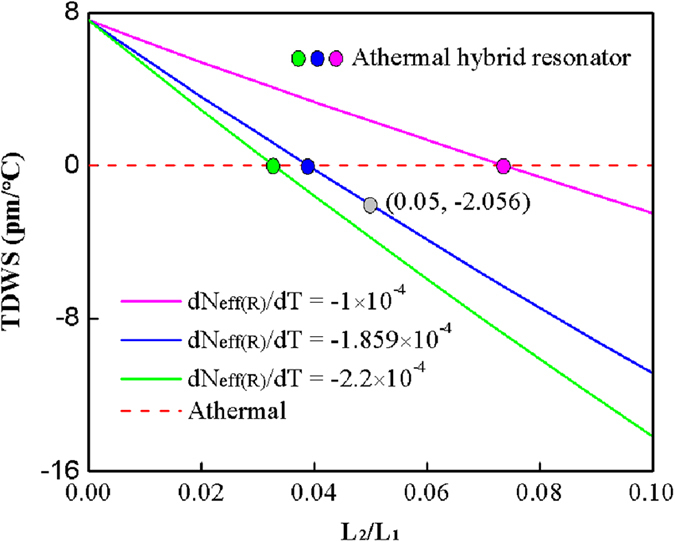
Theoretically calculated TDWSs of hybrid resonators with respect to *dN*_*eff(R*)_/*dT* and *L*_*2*_/*L*_*1*_.

Figure 5 shows the calculated TDWSs of three hybrid resonators with different *dN*_*eff(R*)_/*dT* as a function of *L*_*2*_/*L*_*1*_. It is clear from the solid blue line in Fig. 5 that the fabricated hybrid resonator with *L*_*1*_ = 30 cm, *L*_*2*_ = 1.5 cm, i.e. the ratio regarding *L*_*2*_/*L*_*1*_ is 0.05, has a TDWS of about −2pm/°C. Especially, athermal resonator can be realized when *L*_*2*_/*L*_*1*_ = 0.0389. Compared with the other two TDWS lines, we found the TDWSs have a similar variation trend with respect to *L*_*2*_/*L*_*1*_, i.e., the TDWS linearly decreases from positive to negative with the increasing of *L*_*2*_/*L*_*1*_, and has a zero point to realize athermal resonator. In addition, the zero point of TDWS with respect to *L*_*2*_/*L*_*1*_ increases with the increasing 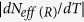. But the TDWSs exhibit different decreasing slope and zero condition. The difference is due to the different offsetting between the negative *dN*_*eff(R*)_/*dT* of LRSPP waveguide and the positive TOC of silica fiber. Furthermore, Fig. 5 also shows that the TDWS of hybrid resonator is determined by the ratio of *L*_*2*_/*L*_*1*_ rather than the size of the resonator.

Compared with the reported ROG resonators including the silica fiber resonator^30^, the glass waveguide resonator^31^, the polymer waveguide resonator^32^, the InP waveguide resonator^33^, the LiNbO_3_ waveguide resonator^34^, the hollow-core photonic bandgap fiber (PBF) resonator^9^ and etc., the hybrid resonator shows a better thermal stability. Generally, the thermal sensitivity of traditional ROG resonators is estimated by 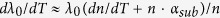
35, where *n* and *dn/dt* are the refractive index and the TOC of waveguide material, *α*_*sub*_ is the TEC of substrate. For the traditional ROG resonators, the TDWS are from about several to several hundred pm/°C. For example, the TDWS of the silica fiber resonator is about 7.6 pm/°C, and that of the polymer resonator is always more than one hundred pm/°C^32^. At present, the ROG resonator based on PBF shows the lowest TDWS of about less than 1 pm/°C^36^. Compared with the reported work, this work gives a ROG resonator with a TDWS of about 2 pm/°C which is smaller than traditional fiber resonators. More importantly, this work presents a pathway to achieve athermal resonator with a better thermal stability than the PBF resonator for ROG.

Given the above, the hybrid resonator has precious properties of single ESOP and temperature insensitivity. Therefore, by using the hybrid resonator, the polarization noise and the temperature noise in RFOG can be effectively eliminated. Then, we estimated the limited sensitivity of RFOG based on hybrid resonator only taking account of the shot noise from the photodiodes included in gyroscope readout system by^32^


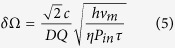


where *c* is the speed of light in vacuum, *D* is diameter of the resonator, *Q* is the quality factor of resonator, *v*_*m*_ is the gyroscope operating frequency, *P*_*in*_ is the light power at photodetector, *h* is the Planck constant, *τ* is the integration time, *η* is the quantum efficiency of the photodiodes. It is clear from Fig. 4(b) that the resonator shows a narrow full width at half maximum (*FWHM*) of about 2.1 pm, and a free spectral range (FSR) of about 5.8 pm. Therefore, the quality factor (*Q*) of the hybrid resonator is about 7.4 × 10^5^ obtained by *Q* = *λ*/*FWHM*. As a result, a shot-noise-limited sensitivity of 4.5°/h for *η* = 0.9, *τ* = 1 s and *P*_*in*_ = 10 mW can be potentially realized.

**Figure 6:**
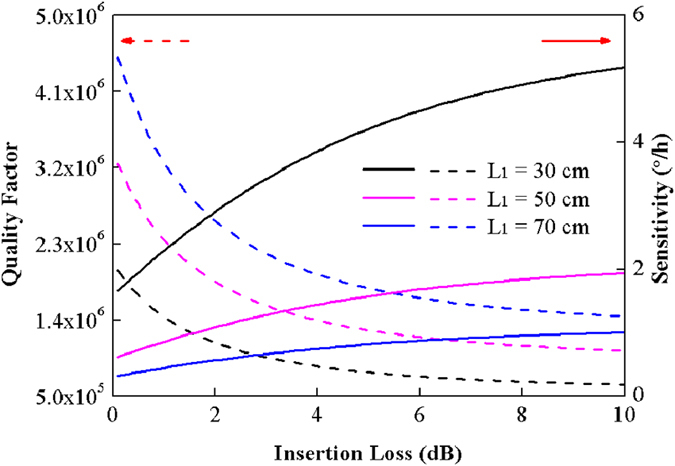
Calculated quality factors of hybrid resonator and the corresponding shot-noise-limited sensitivities of gyroscope as a function of the insertion loss of the LRSPP waveguide coupler at different lengths of silica fiber.

Figure 6 shows the dependence of *Q* factor of hybrid resonator and the corresponding shot-noise-limited sensitivity of RFOG on the length of fiber and the insertion loss of LRSPP waveguide coupler. It is known from Fig. 6 that the *Q* factor and the sensitivity can be promoted by decreasing the insertion loss of LRSPP waveguide coupler and enlarging the resonator size. From the dashed black line in Fig. 6, we know that the LRSPP coupler in the fabricated hybrid resonator with *L*_*1*_ = 30 cm, *Q* = 7.4 × 10^5^ has a corresponding insertion loss of about 5.8 dB. This indicates the LRSPP waveguide in the 1.5-cm-long coupler has a propagation loss of less than 3.87 dB/cm. For the fabricated LRSPP coupler with insertion loss of 5.8 dB, the corresponding *Q* factors of hybrid resonators with *L*_*1*_ = 50 cm and *L*_*1*_ = 70 cm are about 1.21 × 10^6^ and 1.68 × 10^6^, respectively. And the corresponding limited sensitivities of RFOG are about 1.66°/h and 0.86°/h, respectively. Compared with the reported work, this gyroscope sensitivity based on the hybrid resonator is higher than that based on the InP resonator, the polymer resonator and the LiNbO_3_ resonator^32^, but is lower than that based on the silica fiber/waveguide resonator and the PBF resonator^9^,^30^,^37^. However, the ROG based on the hybrid resonator still reaches the level of the rate-grade and tactical-grade gyroscope, and shows wide applications. Furthermore, the sensitivity can be further improved by decreasing the insertion loss of LRSPP coupler and increasing the size of the hybrid resonator.

## Conclusion

In this paper, we demonstrated a novel hybrid resonator consisting of a polymer-based LRSPP coupler and a silica fiber to suppress the polarization noise and the temperature noise in RFOG system. Firstly, we fabricated a LRSPP waveguide coupler and measured its polarization dependence. The results show that the LRSPP coupler only works at TM polarization, while it blocks at TE polarization. We further constructed a hybrid resonator with a 1.5-cm-long LRSPP coupler and a 30-cm-long silica fiber. By measuring the resonance spectrum of hybrid resonator, we found that the fabricated hybrid resonator shows unique single-polarization property. Meanwhile, we also analyzed the temperature dependence of the hybrid resonator. We showed that the TDWS of the hybrid resonator is heavily depended on the length ratio between the LRSPP waveguide coupler and the silica fiber, rather than the size of resonator. Athermal resonator can be even realized with optimal length ratio. Finally, we evaluated the shot noise limited sensitivity of RFOG based on this hybrid resonator. The calculated results show that this RFOG has a potential sensitivity around several degrees per hour. Therefore, this work provides a promising way to realize rate-grade and tactical-grade RFOG without polarization noise and temperature noise.

## Methods

### Fabrication of LRSPP waveguide coupler

Firstly, a silicon chip was cleaned and baked at 90 °C for 3 min. A thin adhesive layer was then spin coated on a silicon wafer, and baked at 110 °C for 3 min. An about 20-μm-thick lower cladding was spin coated on a silicon wafer, and then UV curing was performed in a UV light (365 nm) irradiation chamber with an optical power density of 50 mW/cm^2^ on the wafer for 2 min. The UV curing was performed in the absence of oxygen by flowing nitrogen through the chamber. After the UV curing, the wafer was baked at 160 °C for 30 min. Then a 3-μm-thick negative photoresist of NR9-3000PY was spin coated on the lower layer, baked at 150 °C for 1 min, exposed under 365-nm light with optical power density of 10 mW/cm^2^ for 57 s, post-baked at 100 °C for 1 min and developed in resist developer RD6 by spray or immersion. After these, LRSPP coupler pattern formed by excellent trapezoidal grooves with width of 4 μm was obtained. Then a 10-nm-thick silver film was fabricated by electron beam evaporation with a speed of 0.05 nm/s, and removal of resist was performed in resist remover RR5. Then the silver core of LRSPP waveguide coupler with width of 4 μm and thickness of 10 nm was obtained. Finally, a 20-μm-thick upper cladding was formed by spin coating and was UV-cured with the same conditions as explained previously. For better stability and perfect curing of the polymer film, the whole wafer went through an additional thermal curing process at 160 °C for 60 min.

## Additional Information

**How to cite this article**: Qian, G. *et al*. Hybrid fiber resonator employing LRSPP waveguide coupler for gyroscope. *Sci. Rep.* **7**, 41146; doi: 10.1038/srep41146 (2017).

**Publisher's note:** Springer Nature remains neutral with regard to jurisdictional claims in published maps and institutional affiliations.
